# Significance of Intraoperative Lavage Cytology in Perihilar Cholangiocarcinoma

**DOI:** 10.1002/ags3.70044

**Published:** 2025-06-02

**Authors:** Kentaro Miyake, Ryusei Matsuyama, Yusuke Nakazaki, Kota Sahara, Tomoaki Takahashi, Yutaro Kikuchi, Yasuhiro Yabushita, Yu Sawada, Yuki Homma, Itaru Endo

**Affiliations:** ^1^ Department of Gastroenterological Surgery Yokohama City University Graduate School of Medicine Yokohama Kanagawa Japan

**Keywords:** lavage cytology, perihilar cholangiocarcinoma, peritoneal metastasis

## Abstract

**Background:**

Perihilar cholangiocarcinoma (PHC) has a poor prognosis, with frequent early metastatic recurrence after curative resection. Peritoneal metastasis (PM) is particularly difficult to diagnose and predict. While intraoperative lavage cytology (CY) is a standard method to detect PM, its utility remains unclear in PHC. In this study, we investigated the significance of CY in PHC patients.

**Patients and Methods:**

We retrospectively examined the relationship between CY status and clinicopathological factors in 285 PHC patients and underwent CY for resection between January 1993 and December 2020.

**Results:**

32/285 (11.2%) cases were CY positive. We excluded 61 cases of expiratory laparotomy due to distant metastasis or local extension and nine cases with postoperative hospital mortality. 215 cases were divided into CY positive group (CY+, *n* = 12) and CY negative group (CY−, *n* = 203). PM recurrence was higher in the CY+ group (33.3%) than in the CY− group (13.3%), though not statistically significant (*p* = 0.087). Median recurrence‐free survival was 21.7 months (CY+) versus 30.6 months (CY−) (*p* = 0.357), and early recurrence (< 6 months) occurred in 16.7% versus 10.3% (*p* = 0.552). The median survival time was 42.8 months (CY+) and 44.1 months (CY−), with no significant difference (*p* = 0.678).

**Conclusion:**

CY status was not strongly associated with PM or predictive of PM recurrence. Surgical resection may be justified in CY+ cases, as no statistically significant difference in prognosis was observed; however, these findings are exploratory and require validation in future studies.

## 1 Introduction

Perihilar cholangiocarcinoma (PHC) is a distinct subtype of biliary tract malignancy that arises at the confluence of the right and left hepatic ducts and notorious for its dismal prognosis, with 5‐year survival rates often reported to be 32%–44% [1, 2]. This poor outcome is largely attributable to the tumor's aggressive biological behavior, anatomical complexity, and frequent local invasion of vascular structures. Even after curative resection, the risk of recurrence remains high, ranging from 50% to 70%, emphasizing the need for vigilant postoperative surveillance and adjuvant therapies [3, 4, 5].

Among various recurrence patterns in PHC, peritoneal metastasis (PM) poses a particular challenge. PM often heralds a dismal clinical course due to difficulty in detection, limited surgical options, and poor response to systemic treatments [6]. Several investigations have reported that early postoperative recurrence—within 6–12 months—occurs in a substantial proportion of PHC patients, indicating that microscopic dissemination might already exist at the time of surgery [7]. In particular, the detection of cancer cells in the peritoneal cavity has been linked to worse outcomes in other gastrointestinal and gynecological malignancies [8, 9, 10].

In other gastrointestinal malignancies, such as gastric cancer, intraoperative lavage cytology (CY) obtained via peritoneal lavage is routinely employed to detect occult tumor cells, which has implications for staging and prognosis [11, 12]. Most evidence on the prognostic significance of CY positivity stems from gastric cancer and some colorectal cancer studies [13]. In these tumors, a positive CY finding can alter staging (e.g., categorizing the disease as M1 in certain classification systems) and might prompt changes in clinical management [14].

By contrast, the utility of CY in guiding therapeutic decision‐making for biliary tract cancer including PHC remains less clear [15, 16]. The literature is more sparse, and positive CY has not been uniformly recognized as a definitive indicator of systemic or peritoneal spread. Consequently, there is variability in clinical practice regarding whether to proceed with resection or to offer neoadjuvant therapy when CY positivity is discovered intraoperatively. Specifically, questions persist about whether a positive intraoperative cytology should be considered a contraindication to curative‐intent resection, or whether these patients might still benefit from surgery followed by appropriate adjuvant therapy. Additionally, it is unclear how CY results could guide perioperative management to potentially improve prognoses. Clarifying these uncertainties is critical to improving patient selection and tailoring perioperative management strategies.

In light of the above, the primary objective of this study was to investigate the clinical significance of intraoperative peritoneal cytology in PHC patients. We aimed to evaluate whether CY status correlates with PM, early postoperative recurrence, and overall survival (OS).

## 2 Materials and Methods

### 2.1 Patient Selection

We retrospectively analyzed the records of 285 patients pathologically diagnosed with PHC at the Yokohama City University (YCU) Hospital between January 1993 and December 2020, all of whom underwent intraoperative peritoneal lavage for cytological examination. Specifically, we noted that postoperative adjuvant therapy consisted of oral administration of S‐1 for 6 months following surgical resection. The study protocol was approved by the Ethical Advisory Committee of the YCU and conducted in accordance with the provisions of the Declaration of Helsinki (IRB number: F250400003). An opt‐out approach was employed in lieu of formal written informed consent. Specifically, we posted a document outlining the study's objectives, scope, and data‐handling procedures on our hospital's website and on notice boards within the facility, thus providing patients (or their representatives) an opportunity to decline participation. If no objection was received, we regarded this as implied consent. All patient data were anonymized to ensure privacy, and no information that could identify individual patients was included in the analyses.

### 2.2 The Method of CY and Its Role in the Treatment Strategy

Following laparotomy, the pelvic cavity was irrigated with 100 mL of 0.9% sodium chloride, and the peritoneal lavage fluid was collected for pathological analysis. Smears were prepared from the centrifuged sediment, stained using Papanicolaou and/or Giemsa methods, and evaluated by qualified pathologists. CY+ was characterized by the detection of cancer cells in the peritoneal lavage. The washing cytology was not performed as a rapid test, and its results were not used as a basis for determining the feasibility of resection or the decision to administer adjuvant chemotherapy postoperatively.

### 2.3 Statistical Analysis

All statistical analyses were performed using the EZR (Saitama Medical Center, Jichi Medical University) [17]. For comparison of two groups, a *t*‐test and Pearson's *χ* ^2^ test were utilized. Kaplan–Meier curves illustrated survival times, and the log‐rank test compared groups. Univariate and multivariate analyses using Cox regression were performed to identify prognostic factors. Statistical significance was set at *p* < 0.05.

## 3 Results

### 3.1 Patients' Characteristics

**FIGURE 1:**
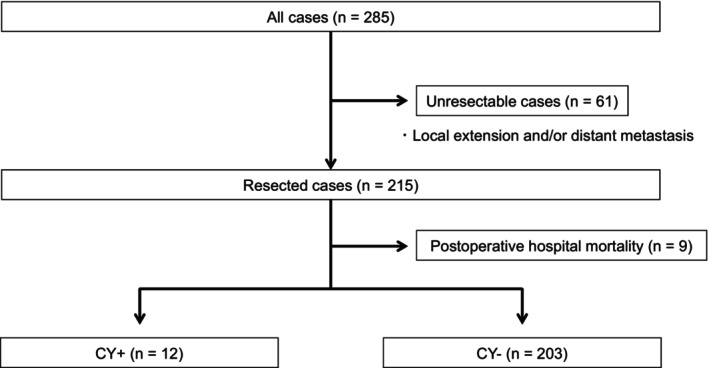
Schema of patient selection. A total of 61 cases were excluded due to local extension and/or distant metastases. Additionally, nine cases with postoperative mortality were excluded. The remaining 215 patients were categorized into two groups: the CY positive group (CY+, *n* = 12) and the CY negative group (CY−, *n* = 203).

All patients underwent laparotomy aiming curative resection. CY positive ratio was 11.2% (32/285). 61 cases were diagnosed as unresectable due to local extension and/or distant metastases (Figure 1). Nine cases with postoperative mortality were also excluded. 215 patients were divided into two groups, considering the CY status. 12 were included into CY positive group (CY+) and 203 were included into CY negative group (CY−). The clinicopathological characteristics of the study population are summarized in Table 1. The analysis included 215 patients, categorized into two groups: CY+ (*n* = 12) and CY− (*n* = 203). The mean age of the CY+ group was 64 ± 13 years, while the CY− group had a mean age of 68 ± 10 years (*p* = 0.157). Gender distribution was comparable between the groups, with a male‐to‐female ratio of 9:3 in the CY+ group and 142:61 in the CY− group (*p* = 1.000).

**TABLE 1:** Clinicopathological characteristics of patients.

Variables		CY+ (*n* = 12)	CY− (*n* = 203)	*p*
Age		64 ± 13	68 ± 10	0.157
Gender	M/F	9/3	142/61	1.000
CEA	ng/mL	3.85 (1.25–9.15)	2.40 (1.60–3.90)	0.313
CA19‐9	U/mL	43 (19.5–369)	36.5 (14–105.8)	0.400
ICGR15	%	11.41 (8.94–13.40)	12.15 (8.70–15.64)	0.720
Bismuth type4		2 (16.7%)	50 (24.6%)	0.735
Preoperatove biliary drainage		9 (75%)	175 (86.2%)	0.436
Perctaneous drainage		3 (25%)	33 (16.3%)	0.417
Clinical T factor	≥ 3	11 (91.7%)	160 (78.9%)	0.467
Clinical N factor	+	8 (66.7%)	105 (51.7%)	0.382
Neoadjuvant chemotherapy		7 (58.3%)	85 (41.9%)	0.240
Portal vein embolization		5 (41.7%)	101 (49.8%)	0.576
Surgical procedure				
Trisectionectomy/Hemihepatectomy/HPD/Others		1/8/1/2	25/134/25/19	0.943
Vascular resection		8 (66.7%)	120 (59.1%)	0.782
Operation time	min	782 (728–913)	774 (665–909)	0.546
Blood loss	mL	1503 (1060–2057)	1362 (908–2192)	0.711
Transfusion		6 (50%)	107 (52.7%)	1.000
Clavien‐Dindo classification ≥ IIIa		4 (33.3%)	98 (48.2%)	0.382
Pathological T category	≥ 3	6 (50.0%)	88 (43.3%)	0.126
Pathological N category	+	4 (33.3%)	87 (42.9%)	0.762
Lymphatic invasion		6 (50%)	75 (36.9%)	0.554
Venous invasion		8 (66.7%)	101 (49.8%)	0.400
Perineural invasion		10 (83.3%)	134 (66.1%)	0.388
R0 resection		11 (91.7%)	150 (73.9%)	0.302
Hospitalization	Days	18 (14–25)	27 (18–42)	0.016
Adjuvant chemotherapy		7 (58.3%)	102 (50.2%)	0.768

Serum carcinoembryonic antigen (CEA) levels were higher in the CY+ group (median: 3.85, interquartile range [IQR]: 1.25–9.15) compared to the CY− group (median: 2.40, IQR: 1.60–3.90); however, the difference was not statistically significant (*p* = 0.313). Similarly, carbohydrate antigen 19–9 (CA19‐9) levels were elevated in the CY+ group (median: 43, IQR: 19.5–369) compared to the CY− group (median: 36.5, IQR: 14–105.8), but this difference also did not reach statistical significance (*p* = 0.400). There were no significant differences between the CY+ and CY− groups in terms of preoperative biliary drainage (75% vs. 86.2%, *p* = 0.436). Among these, percutaneous drainage was used in 25% and 16.3% of patients, respectively (*p* = 0.417). Except for a shorter postoperative hospital stay observed in the CY− group, there were no significant differences in perioperative factors between the two groups. The proportion of patients who received postoperative adjuvant chemotherapy was also comparable between the CY+ and CY− groups (58.3% vs. 50.2%, *p* = 0.768).

### 3.2 Survival Analysis Considering CY Status

**FIGURE 2:**
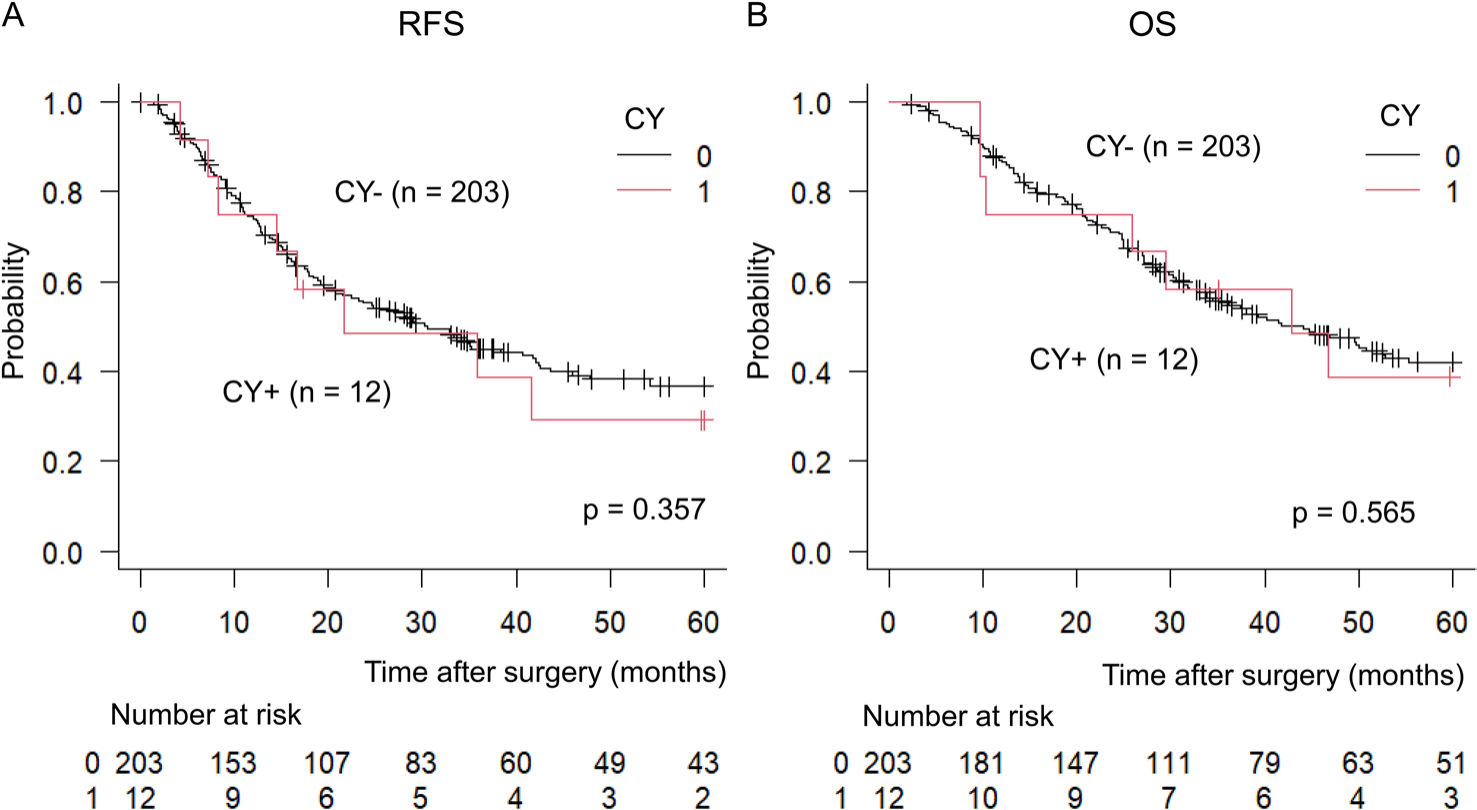
Kaplan–Meier Analysis of Survival Outcomes. (A) Relapse‐free survival (RFS) between the CY+ and CY− groups. The median RFS was 21.7 months in the CY+ group and 30.6 months in the CY− group, showing a favorable trend in the CY− group, although the difference was not statistically significant (*p* = 0.357). (B) Overall survival (OS) between the CY+ and CY− groups. The median survival time (MST) was 42.8 months in the CY+ group and 44.1 months in the CY− group, with no statistically significant difference observed (*p* = 0.565).

Figure 2 illustrated the Kaplan–Meier analysis focusing on the relapse‐free survival (RFS) and overall survival between the two groups. The median RFS was 21.7 months in the CY+ group and 30.6 months in the CY− group, showing a favorable trend in the CY− group but no statistically significant difference (*p* = 0.357) (Figure 2A). There was no significant difference regarding OS, with 42.8 months in the CY positive group and 44.1 months in the CY negative group for MST (*p* = 0.565) (Figure 2B).

### 3.3 Details of Recurrence in Both Groups

The data presented in Table 2 provide the characteristics of recurrence in two groups, CY+ (*n* = 12) and CY− (*n* = 203). In the CY+ group, 10 out of 12 individuals (83.3%) experienced recurrence, while 120 out of 203 individuals (59.1%) in the CY− group experienced recurrence. The difference between the groups was not statistically significant (*p* = 0.246). In the CY+ group, 2 out of 12 individuals (16.7%) had recurrence within 6 months after the operation, compared to 21 out of 203 individuals (10.3%) in the CY− group. This difference was also not statistically significant (*p* = 0.629). Regarding PM, a trend toward a higher rate was observed in the CY+ group (33.3%) compared to the CY− group (13.3%), although no statistically significant difference was found. Similarly, no significant differences were observed in other recurrence patterns, including local recurrence, hepatic metastasis, pulmonary metastasis, and bone metastasis.

**TABLE 2:** Characteristics of the recurrence in two groups.

Variables	CY+ (*n* = 12)	CY− (*n* = 203)	*p*
Recurrence	10 (83.3%)	120 (59.1%)	0.246
Recurrence within 6 months after operation	2 (16.7%)	21 (10.3%)	0.629
Pattern of recurrence			
Local	1 (8.3%)	46 (22.7%)	0.310
Lymph node	2 (16.7%)	29 (14.3%)	0.697
Peritoneum	4 (33.3%)	27 (13.3%)	0.087
Liver	3 (25%)	35 (17.2%)	0.466
Lung	2 (16.7%)	12 (5.9%)	0.190
Bone	1 (8.3%)	4 (2.0%)	0.260
Multiple sites	3 (25%)	35 (17.2%)	0.466

### 3.4 Univariate and Multivariate Analysis of the Prognostic Factors for OS

In univariate analysis, pT3 or higher (HR 1.933 (1.364–2.740), *p* < 0.001), positive lymph node metastasis (HR 2.443 (1.722–3.468), *p* < 0.001), venous invasion (HR 1.798 (1.268–2.550), *p* < 0.001), perineural invasion (HR 1.843 (1.249–2.720), *p* = 0.002), and residual tumor (HR 1.933 (0.639–1.277), *p* < 0.001) were significant prognostic factors (Table 3). CY status and the presence or absence of postoperative adjuvant chemotherapy were not statistically significant prognostic factors. In multivariate analysis, positive lymph node metastasis (HR 2.124 (1.482–3.045), *p* < 0.001), venous invasion (HR 1.488 (1.020–2.169), *p* = 0.039), and residual tumor were significant prognostic factors (HR 1.604 (1.109–2.319), *p* = 0.012).

**TABLE 3:** Univariate and multivariate analyses of factors associated with overall survival in PHC patients.

Variables	Univariate analysis	*p*	Multivariate analysis	*p*
CY+	HR 1.154 (0.586–2.274)	0.678		
pT ≥ 3	HR 1.933 (1.364–2.740)	< 0.001	HR 1.444 (0.998–2.090)	0.051
pN+	HR 2.443 (1.722–3.468)	< 0.001	HR 2.124 (1.482–3.045)	< 0.001
ly+	HR 1.387 (0.974–1.976)	0.069		
v+	HR 1.798 (1.268–2.550)	< 0.001	HR 1.488 (1.020–2.169)	0.039
ne+	HR 1.843 (1.249–2.720)	0.002		
R1 or R2	HR 1.933 (0.639–1.277)	< 0.001	HR 1.604 (1.109–2.319)	0.012
AC	HR 0.904 (0.639–1.277)	0.565		

### 3.5 Details of Clinical Course in the CY+ Group

Table 4 showed the summary of clinical characteristics and postoperative course of CY+ patients. The mean age was 64 years, with a male‐to‐female ratio of 9:3. Among the 12 patients, 7 (58%) underwent NAT. The surgical procedures included lobectomy or more extensive resection in nine (75%) patients, segmentectomy in one patient, and extrahepatic bile duct resection in two patients. Postoperative adjuvant chemotherapy was administered to 7 of the 12 patients. Although 9 of the 12 patients experienced recurrence, only 3 (25%) recurred within 1 year, and peritoneal dissemination was observed in only 3 patients.

**TABLE 4:** Clinical characteristics and postoperative course of CY+ patients.

Case	Age	Gender	NAT	Procedure	AC	Recurrence	Pattern of recurrence	Prognosis	RFS	OS
1	66	M	0	Left hemihepatectomy + C	0	1	Lymph node	DOD	14.4	25.9
2	62	F	1	Extrahepatic bile duct resection	1	1	PM	AWD	112.9	174.3
3	73	M	0	Right hemihepatectomy + C	0	1	Multiple	DOD	4.2	9.7
4	72	M	0	Right hemihepatectomy + C	1	1	Local	DOD	35.9	70.1
5	83	M	1	Extrahepatic bile duct resection	1	0	—	NED	59.8	59.8
6	43	M	0	Left hemihepatectomy + C	0	1	Lung	DOD	8.3	10.3
7	63	M	1	Right hemihepatectomy + C	0	1	Multiple	DOD	41.6	46.8
8	71	M	1	Extended left hemihepatectomy + C	1	1	Liver	DOD	16.6	29.5
9	77	M	0	Extended right hemihepatectomy + C	0	0	—	DOO	110.5	111.2
10	49	F	1	Left trisectionectomy + C	1	1	Multiple (PM+)	DOD	21.7	42.8
11	68	F	1	Right hemihepatectomy + C + PD	1	1	PM	DOD	7.1	9.6
12	41	M	1	Anterior sectionectomy + C	1	0	—	NED	17.3	35.0

Abbreviations: AWD, alive with disease; DOD, dead of disease; DOO, dead of other disease; NAT, neoadjuvant therapy; NED, no evidence disease.

## 4 Discussion

This study evaluated the clinical significance of CY in predicting PM and overall prognosis in patients undergoing curative‐intent resection for PHC. Although the CY+ group showed a higher incidence of PM recurrence (33.3% vs. 13.3% in CY−), the difference was not statistically significant (*p* = 0.087). No significant differences were observed in RFS, OS, or early recurrence within 6 months between the two groups. Multivariate analysis also indicated that CY status was not a prognostic factor for OS. These findings suggest that CY status alone does not reliably predict PM or impact long‐term outcomes in PHC patients.

This study is one of the largest retrospective analyses specifically assessing the prognostic value of intraoperative lavage CY in PHC. Unlike previous reports that included a heterogeneous mix of biliary tract cancers, this study focused exclusively on PHC, thus providing more specific insights relevant to this disease entity. Given that PHC differs markedly from intrahepatic or distal cholangiocarcinoma in terms of anatomical complexity, patterns of tumor spread, and surgical approach, analyzing PHC as a separate and homogeneous cohort enhances the clinical applicability and interpretability of our findings in this unique subset of biliary tract cancer. Another notable strength is the long study period, allowing for robust survival analyses. The extensive dataset facilitated comprehensive multivariate analysis, adjusting for potential confounders such as lymph node metastasis, vascular invasion, and perineural invasion.

The finding that CY positivity did not significantly influence RFS or OS is novel and challenges the conventional assumption that CY positivity invariably correlates with poor prognosis. This suggests that surgical resection could still be justified in selected CY+ patients, potentially expanding treatment options for this high‐risk group. This insight is particularly relevant given the high recurrence rates observed in PHC even after curative‐intent surgery, emphasizing the need for more refined prognostic tools.

Our findings are consistent with the previous report, which also reported no significant difference in OS between CY+ and CY− groups following primary tumor resection in PHC patients [18]. In their study, the CY+ resected group exhibited similar survival to the CY− resected group and significantly better survival than the CY+ unresected group, suggesting that CY positivity alone should not be a contraindication for surgical resection. Similarly, the high administration rate of postoperative therapy in the CY+ group likely contributed to the comparable survival outcomes, as observed in our cohort. Moreover, Matsukuma et al. demonstrated that CY positivity was not an independent prognostic factor for biliary tract cancer, although it was associated with a higher incidence of peritoneal dissemination [19]. These studies support our observation that CY status alone is insufficient to guide clinical decision‐making.

In contrast, CY positivity is a well‐established poor prognostic factor in gastric and pancreatic cancers, influencing staging and treatment strategies [9, 20, 21]. In gastric cancer, CY positivity is classified as M1 disease according to the Japanese Gastric Cancer Association guidelines, which often precludes curative surgery [22]. Likewise, in pancreatic cancer, positive CY is associated with worse OS and is considered an indicator of micrometastasis [23]. This discrepancy between gastrointestinal malignancies and PHC suggests potential differences in tumor biology, peritoneal dissemination mechanisms, or immune microenvironments.

Several reasons could explain this discrepancy. PHC is known for its local invasiveness and propensity for lymphatic rather than peritoneal dissemination [1, 3, 24, 25], whereas gastric and pancreatic cancers more frequently disseminate to the peritoneum. Additionally, the anatomical location of PHC at the biliary confluence might limit direct peritoneal spread compared to the stomach or pancreas, which are in close proximity to the peritoneal cavity [26].

Recent studies have also suggested that the clinical significance of CY positivity may depend on the method of detection, with cell‐free DNA showing higher sensitivity than conventional cytology [27]. It has also been reported that the results of cytology may vary depending on the site of specimen collection [28]. This raises the possibility that conventional CY may underestimate the presence of peritoneal dissemination, potentially explaining the lack of prognostic impact observed in our study.

The lack of a statistically significant impact of CY positivity on PM, RFS, and OS in this study could be attributed to several factors. First, the relatively low rate of CY positivity (6.0%) limits the statistical power to detect subtle differences. Second, the non‐significant trend toward higher PM recurrence in the CY+ group suggests that CY positivity might indicate minimal peritoneal dissemination below the detection threshold of current imaging modalities [29, 30]. However, the absence of significant differences in survival outcomes implies that such microscopic dissemination does not necessarily translate into overt PM or compromised prognosis.

This study has several limitations. The retrospective design inherently carries the risk of selection bias. Although multivariate analysis was performed to adjust for potential confounders, unmeasured variables may have influenced the outcomes. Additionally, the study was conducted at a single center, which may limit the generalizability of the results. The relatively small number of CY+ patients further limits the statistical power to detect differences in survival outcomes. Furthermore, the CY method used in this study was not standardized as a rapid test, which may have led to variability in interpretation and clinical application. Lastly, the lack of molecular or genomic data precludes an understanding of the underlying biological mechanisms driving the observed clinical behavior of CY+ PHC. Future multicenter prospective studies with standardized CY protocols and molecular analyses are needed to validate these findings and explore the biological significance of CY positivity.

## 5 Conclusion

Our investigation indicates that positive peritoneal cytology, in the context of an otherwise resectable disease, does not necessarily preclude curative‐intent surgery or diminish long‐term survival when appropriately managed with a multidisciplinary treatment approach. Although caution is warranted due to the small sample size of CY+ patients, the comparable OS and only partially elevated recurrence rate suggest that these patients can still benefit from surgical resection and adjunct therapies. These findings have important implications for clinicians and policymakers in guiding treatment decisions and resource allocation, highlighting that CY positivity alone should not deter consideration of potentially curative interventions.

## Author Contributions

**Kentaro Miyake:** conceptualization, writing – original draft, data curation, methodology, formal analysis, project administration. **Ryusei Matsuyama:** conceptualization, methodology. **Yusuke Nakazaki:** data curation, project administration, investigation. **Kota Sahara:** formal analysis. **Tomoaki Takahashi:** writing – review and editing. **Yutaro Kikuchi:** writing – review and editing. **Yasuhiro Yabushita:** writing – review and editing. **Yu Sawada:** writing – review and editing. **Yuki Homma:** writing – review and editing. **Itaru Endo:** conceptualization, methodology, writing – review and editing, supervision.

## Ethics Statement

The study protocol was approved by the Ethical Advisory Committee of Yokohama City University Graduate School of Medicine (approval number: B200700094) and the Institutional Review Board of the participating hospital. An opt‐out method was used after disclosure of the study information instead of written informed consent.

## Conflicts of Interest

The authors declare no conflicts of interest. Itaru Endo is an editorial board member of Annals of Gastroenterological Surgery.
